# Identification and characterization of the novel Col10a1 regulatory mechanism during chondrocyte hypertrophic differentiation

**DOI:** 10.1038/cddis.2014.444

**Published:** 2014-10-16

**Authors:** J Gu, Y Lu, F Li, L Qiao, Q Wang, N Li, J A Borgia, Y Deng, G Lei, Q Zheng

**Affiliations:** 1 Department of Hematology and Hematological Laboratory Science, School of Medical Science and Laboratory Medicine, Jiangsu University, Zhenjiang, China; 2 Department of Anatomy and Cell Biology, Rush University Medical Center, Chicago, IL, USA; 3 Department of Pathophysiology, Anhui Medical University, Hefei, China; 4 Department of Pathology and Department of Biochemistry, Rush University Medical Center, Chicago, IL, USA; 5 Department of Internal Medicine and Biochemistry, Rush University Medical Center, Chicago, IL, USA; 6 Department of Orthopaedic Surgery, Xiangya Hospital, Central South University, Changsha, China

## Abstract

The majority of human skeleton develops through the endochondral pathway, in which cartilage-forming chondrocytes proliferate and enlarge into hypertrophic chondrocytes that eventually undergo apoptosis and are replaced by bone. Although at a terminal differentiation stage, hypertrophic chondrocytes have been implicated as the principal engine of bone growth. Abnormal chondrocyte hypertrophy has been seen in many skeletal dysplasia and osteoarthritis. Meanwhile, as a specific marker of hypertrophic chondrocytes, the type X collagen gene (*COL10A1*) is also critical for endochondral bone formation, as mutation and altered *COL10A1* expression are often accompanied by abnormal chondrocyte hypertrophy in many skeletal diseases. However, how the type X collagen gene is regulated during chondrocyte hypertrophy has not been fully elucidated. We have recently demonstrated that Runx2 interaction with a 150-bp mouse *Col10a1* cis-enhancer is required but not sufficient for its hypertrophic chondrocyte-specific reporter expression in transgenic mice, suggesting requirement of additional *Col10a1* regulators. In this study, we report *in silico* sequence analysis of this 150-bp enhancer and identification of its multiple binding factors, including AP1, MEF2, NFAT, Runx1 and TBX5. Using this enhancer as bait, we performed yeast one-hybrid assay and identified multiple candidate *Col10a1*-interacting genes, including *cyclooxygenase 1* (*Cox-1*) and *Cox-2*. We have also performed mass spectrometry analysis and detected EF1-alpha, Fus, GdF7 and Runx3 as components of the specific complex formed by the cis-enhancer and nuclear extracts from hypertrophic MCT (mouse chondrocytes immortalized with large T antigen) cells that express *Col10a1* abundantly. Notably, some of the candidate genes are differentially expressed in hypertrophic MCT cells and have been associated with chondrocyte hypertrophy and Runx2, an indispensible *Col10a1* regulator. Intriguingly, we detected high-level Cox-2 expression in hypertrophic MCT cells. Electrophoretic mobility shift assay and chromatin immunoprecipitation assays confirmed the interaction between Cox-2 and *Col10a1* cis-enhancer, supporting its role as a candidate *Col10a1* regulator. Together, our data support a Cox-2-containing, Runx2-centered *Col10a1* regulatory mechanism, during chondrocyte hypertrophic differentiation.

The human skeleton is divided into two main groups: the axial (skull, vertebral column and ribs) and the appendicular (shoulder girdles and limb long bones) skeleton. Most of the skeleton develops through a pathway called endochondral ossification, which involves a cartilage intermediate. In this pathway, the mesenchymal cells condense and form chondrocytes, chondrocytes continue to differentiate, proliferate and enlarge into hypertrophic chondrocytes. Hypertrophic chondrocytes have been implicated as the principal engine and master regulatory cells for bone growth, as these cells are associated with blood vessel invasion and are surrounded by a calcified extracellular matrix that favors endochondral ossification.^1, 2, 3^

The type X collagen gene (*COL10A1*) is specifically expressed by hypertrophic chondrocytes. As a major component of hypertrophic zone, type X collagen influences deposition of other matrix molecules to this region and thereby provide a proper environment for hematopoiesis, mineralization and modeling that are essential for endochondral ossification.^4,5^ Mutations and abnormal expression of *COL10A1* are closely linked to abnormal chondrocyte hypertrophy that has been seen in multiple skeletal dysplasia and osteoarthritis.^6, 7, 8, 9, 10, 11, 12, 13, 14, 15^ Schmid metaphyseal chondrodysplasia (SMCD) is an autosomal dominantly inherited skeletal disorder caused by human *COL10A1* mutation. SMCD is characterized by short stature, coxa vara and an irregular growth plate, suggesting defective endochondral bone formation, whereas *Col10a1* null mice have compressed growth plate similar to human SMCD.^6, 16, 17, 18^ Cleidocranial dysplasia (CCD) is another autosomal dominant skeletal dysplasia caused by haploinsufficiency of RUNX2, a known master transcription factor (TF) for osteoblast differentiation and chondrocyte maturation.^19, 20, 21, 22, 23, 24^ We have previously shown that CCD has defective endochondral ossification (short stature and cone epiphyses) with decreased *COL10A1* expression and markedly diminished hypertrophic zone.^11^ As to the correlation of *COL10A1* expression and chondrocyte hypertrophy with osteoarthritis, it was previously reported that human osteoarthritic cartilage showed upregulated *COL10A1* expression and enhanced chondrocyte hypertrophy, whereas mesenchymal stem cells from patients with osteoarthritis constitutively express type X collagen.^7,8,25,26^

The above findings demonstrate the significant clinical relevance of collagen X expression with skeletal development and disease. Previously, extensive studies have identified multiple TFs or signaling pathways that contribute to type X collagen gene regulation. These include TFs AP1, carbonic anhydrase (CA) IX, GADD45beta, HIF-2a, MEF-2C, PTH/PTHrP, SOX9, SP3/SP1 and the Bmp/Ihh/Wnt signaling pathways.^13,27, 28, 29, 30, 31, 32, 33, 34, 35^ Notably, most of these factors interact with the proximal promoter of type X collagen gene. We have been working on murine *Col10a1* gene regulation and have demonstrated that Runx2 contributes to hypertrophic chondrocyte-specific *Col10a1*/reporter expression *in vivo* through interaction with both the proximal promoter and the distal enhancer element (150-bp, −4296 to −4147).^36, 37, 38^ However, our data also suggest that other regulators, in addition to Runx2, are needed to specify *Col10a1* promoter activity. In this paper, we report identification and characterization of these regulators by dissection of the 150-bp enhancer using comprehensive bioinformatics, proteomics and transgenic (TG) approaches.

## Results

### Col10a1 enhancer *versus* nonspecific promoter element in TG studies

**Figure 1:**
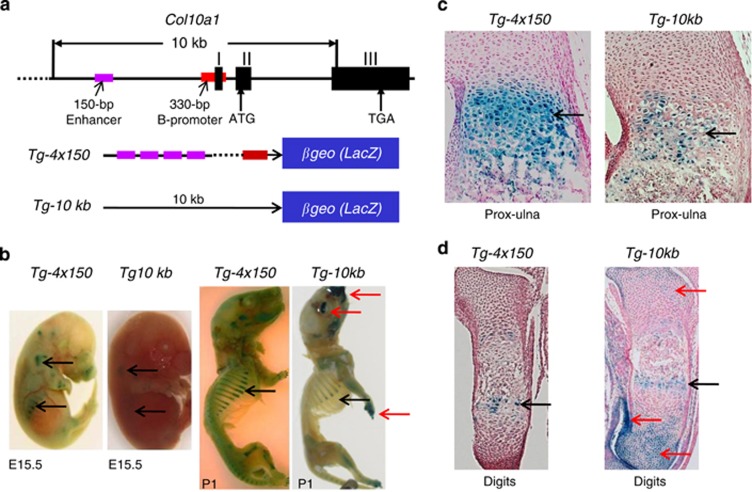
Detailed analysis of *Tg-4x150* and *Tg-10 kb* TG reporter mice. (**a**) Top panel displays *Col10a1* gene structure and the 10 -kb promoter and intronic element. Positions of the 150-bp (−4296 to −4147 bp) *Col10a1* cis-enhancer (purple bar) and its 330-bp (−220 to +110 bp) basal promoter (red bar) are shown. Bottom panel shows the different *Col10a1* promoter/enhancer element to drive the *LacZ* reporter gene. (**b**) X-gal staining and comparison between TG mice *Tg-4x150* and *Tg-10 kb* at both E15.5 (left panel) and P1 (right panel) stages. Much stronger and more specific staining was detected in *Tg-4x150* mice, whereas *Tg-10 kb* mice show extra nonspecific staining in digits and craniofacial region (red arrows). (**c**) Histochemical analysis of the limb sections (proximal ulna) confirmed much stronger blue staining in the hypertrophic zone of *Tg-4x150* mice than in *Tg-10* kb mice (black arrows). (**d**) Analysis of the digit sections detect extra nonspecific blue staining in resting chondrocytes of *Tg-10 *kb mice (red arrows), but not in *Tg-4x150* mice

We have previously generated multiple TG mouse lines using different *Col10a1* promoter and enhancer elements to drive the *LacZ* gene. Varied levels of *LacZ* reporter expression (shown by X-gal blue staining intensity) was observed in these TG mice.^36,37^ We have performed detailed analysis of mice from the *Tg-4x150* and the *Tg-10* kb TG lines. The *Tg-4x150* construct contains only 150-bp *Col10a1* cis-enhancer (four copies) and the basal promoter, whereas the *Tg-10* kb contains both the enhancer and large promoter/intron elements to drive the *LacZ* gene (Figure 1a). The results showed that the *Tg-4x150* mice drive much stronger reporter activity exclusively in hypertrophic chondrocytes than that of *Tg-10* kb mice as demonstrated by whole embryo staining at both E15.5 and P1 stages (Figure 1b) and by histochemical analysis of the limb sections (Figure 1c). There was nonspecific blue staining in resting chondrocytes of the craniofacial and digits of the *Tg-10 *kb mice, but not in *Tg-4x150* mice (Figures 1b and d). These results show that multiple copies of the enhancer (150-bp *Col10a1* distal promoter) mediates higher level and cell-specific reporter activity, whereas the 10-kb *Col10a1* promoter/intronic element contains nonspecific regulatory elements, in addition to the 150-bp enhancer.

### Predictive candidate genes

**Figure 2:**
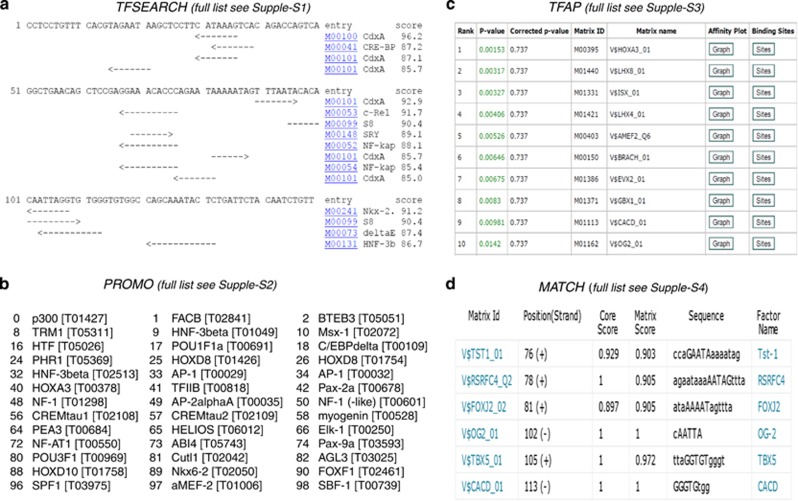
Bioinformatics prediction of TFBS within 150-bp *Col10a1* cis-enhancer. (**a**) TFSEARCH with a cutoff score of 85 identified 15 putative TFBSs including CdxA (six times), C-Rel, S8 (twice) and Nkx-2 etc (see also Supplementary Data S1). (**b**) Promo 3.0 program (with 85 similarity) identified approximately 200 putative TFs, including many AP1s (c-fos, c-Jun, ATF and JDP families), Hif and *α*MEF-2 (see also Supplementary Data S2). (**c**) TRAP identified approximately 50 TFs that showed a *P*-value <0.05, including Hoxa3, CACD and *α*MEF-2 (see also Supplementary Data S3). (**d**) The MATCH program (with a matrix score of 90) identified six putative TFs including Tst-1, RSRFC4, FOXJ2, OG-2, TBX5 and CACD (see also Supplementary Data S4)

We used multiple web-based programs to search for transcription factor binding sites (TFBSs) within the 150-bp enhancer. The TFSEARCH program identified 16 putative *Col10a1*-interacting factors with a cutoff score of 85, whereas only 6 putative TFBSs are shown (CdxA x 2, C-Rel, S8 x 2 and Nkx-2) when the cutoff score was increased to 90 (Figure 2a, Supplementary Data S1).^39,40^ Nearly 200 putative TFs were predicted to bind this cis-enhancer using the Promo 3.0 and TRANSFAC database (version 8.3).^41,42^ These factors include those identified by TFSEARCH and many AP1s (c-fos, c-Jun, ATF and JDP families), Hif and *α*MEF-2 (Figure 2b, Supplementary Data S2). We also use the TRAP software to predict putative binding factors.^43^ TRAP provides affinity-based ranking of TFs with a *P*-value. We identified approximately 50 TFs that showed a *P*-value <0.05, including Hoxa3, CACD and *α*MEF-2 (Figure 2c, Supplementary Data S3). Finally, the MATCH program identified six putative TFs (Tst-1, RSRFC4, FOXJ2, OG-2, TBX5 and CACD) by searching the TRANSFAC database (updated version 10.1, BIOBASE) for TFBS with a matrix score of 90 (Figure 2d, Supplementary Data S4).^44,45^ It is not surprising that the short AT-rich TFs (such as CdxA site: CATAAAG) were identified by multiple programs. Intriguingly, some developmentally important genes, including MEF-2s, Pax family members and Tbx5, were also identified by above dual or triple programs (Figure 2 and Supplementary Data S1–S4).

### Candidate genes identified by yeast one-hybrid (Y1H) screening

**Figure 3:**
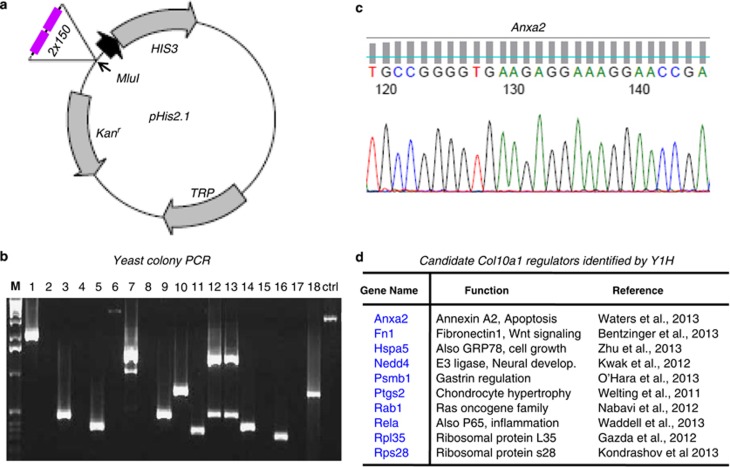
Y1H screening using the 150-bp enhancer as a bait. (**a**) The 150-bp *Col10a1* cis-enhancer (tandem copies, 2 × 150) was cloned upstream of the nutritional reporter gene *HIS3* in a pHis2.1 vector backbone (Clontech). (**b**) After screening a cDNA library derived from hypertrophic MCT cells, approximately 50 yeast colonies grown in selective medium and optimal 3-AT concentration were selected and subjected to PCR amplification. (**c**) Representative sequencing result of the PCR product shows partial sequence of exon II of gene *Anxa2*. (**d**) A list of candidate genes identified by Y1H were shown. These genes include *Fn1*, *Hspa5*, *Nedd4*, *Psmb1*, *Ptgs2*, *Rab1*, *Rela*, *Rpl35* and *Rps28*. The functional description and relevant literatures were as listed

Tandem copies of the 150-bp enhancer elements were successfully cloned into the pHis2.1 vector (Clontech, Mountain View, CA, USA; K1617-1) upstream of the nutritional reporter *HIS3* (Figure 3a). This reporter construct was used as a bait to screen the cDNA library derived from hypertrophic MCT (mouse chondrocytes immortalized with large T antigen) cells by co-transformation with pGADT7-Rec2 expression vector in the Y187 yeast competent cells. Approximately, 50 yeast colonies grown in selective medium and optimal 3-AT concentration were selected and subjected to PCR and sequencing (Figures 3b and c). After searching with the NCBI database, we identified multiple candidate *Col10a1*-interacting genes as listed (*Anxa2*, *Fn1*, *Hspa5*, *Nedd4*, *Psmb1*, *Ptgs2*, *Rab1*, *Rela*, *Rpl35* and *Rps28*). The putative function of these genes and the relevant literatures are as summarized (Figure 3d).

### Candidate genes identified by mass spectrometry

**Figure 4:**
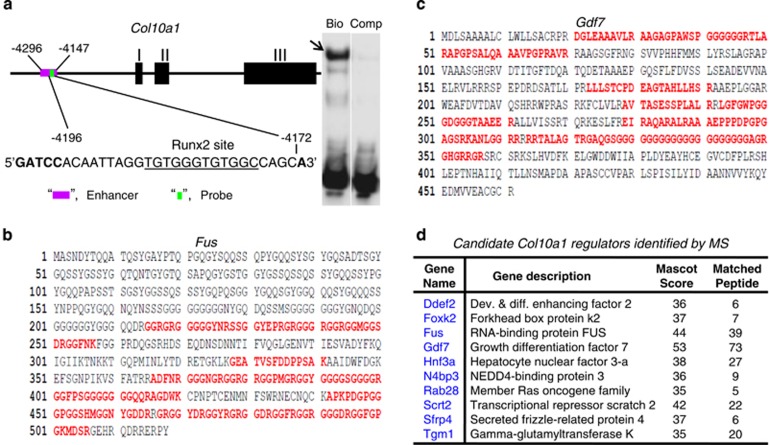
Mass spectrometry analysis of specific DNA/protein complex. (**a**) Position and sequence of the DNA probe from the enhancer region are shown.^38^ Bio-tin labeled cis-element containing the Runx2 site (underlined) can form specific complex with hypertrophic MCT cell nuclear extracts (arrow), whereas no binding complex was observed when using the unlabeled competitive control. (**b**) Fus was identified as it met the criteria for protein identification, that is MASCOT >=35 with >=5 matched peptides (red letters). (**c**) Gdf7 also meets the criteria for protein identification. (**d**) Partial list of genes that encode proteins and meet the criteria for protein identification. These include genes *Ddef2*, *Foxk2*, *Fus*, *Gdf7*, *Hnf3a*, *N4bp3*, *Rab28*, *Scrt2*, *Sfrp4* and *Tgm1*. Gdf7 has the highest (53) MASCOT score with the most (73) matched peptides

We have previously shown that a short DNA element (−4196 to −4172 bp) from the *Col10a1* cis-enhancer can form specific binding complex with hypertrophic MCT cell nuclear extracts (Figure 4a).^38^ We have performed tandem mass spectrometry analysis on above binding mixture and identified >90 putative factors after searching with the SwissProt 51.6 (protein) database. These factors meet the criteria for protein identification that reaches a minimum MASCOT score of 35 with at least 5 matched peptides. Illustrated are Fus (a RNA-binding protein) and Gdf7 (growth differentiation factor 7) that are potential components of the binding complex (Figures 4b and c). Candidate factors with certain MASCOT score and matched peptides are as listed (Figure 4d, Supplementary Data S5). These include genes *Ddef2*, *Foxk2*, *Fus*, *Gdf7*, *Hnf3a*, *N4bp3*, *Rab28*, *Scrt2*, *Sfrp4* and *Tgm1* etc.

### Expression analysis of candidate genes

**Figure 5:**
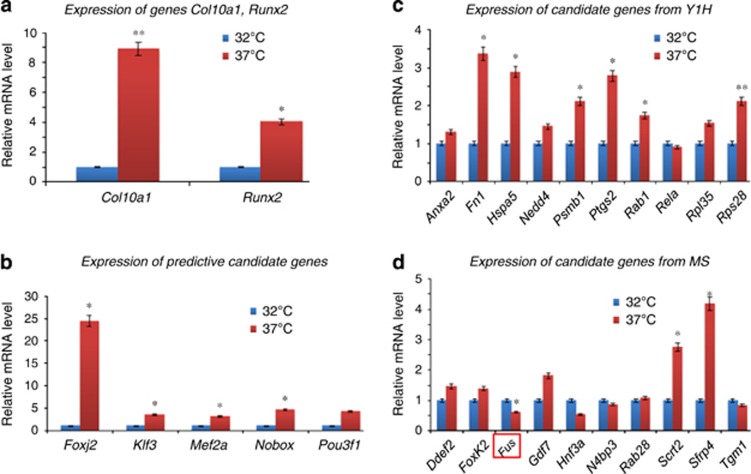
Expression analysis of candidate genes in MCT cells. (**a**) qRT-PCR was performed to examine the mRNA transcript level of marker genes *Col10a1* and *Runx2* in MCT cells. As illustrated, both *Col10a1* (8.8-fold, *P*=0.009) and *Runx2* (4.0-fold, *P*=0.04) are significantly upregulated in hypertrophic MCT cells compared with that in proliferative MCT cells. *P<0.05; **P<0.01. (**b**) Five candidate genes (*Foxj2, Klf3, Mef2a, Nobox* and *Pou3f1*) identified by Match program were examined. Except for *Pou3f1* (3.9-fold, *P*=0.06), all other genes examined show significant upregulation in hypertrophic MCT cells. *P<0.05. (**c**) Ten candidate genes identified by Y1H were selected and examined. Genes *Fn1*, *Hspa5*, *Psmb1*, *Ptgs2*, *Rab1* and *Rps28* are significant upregulated in hypertrophic MCT cells, while others (*Anxa2*, *Nedd4*, *Rela* and *Rpl35*) showed no difference between hypertrophic and proliferative MCT cells. *P<0.05; **P<0.01. (**d**) Ten candidate genes identified by MS were also selected and examined. Genes Scrt2 (2.8-fold, *P*=0.024) and Sfrp4 (4.2-fold, *P*=0.029) showed significant upregulation, whereas *Fus* was significantly downregulated (0.6-fold, *P*=0.041) in hypertrophic MCT cells. Genes (*Ddef2*, *FoxK2*, *Gdf7*, *Hnf3a*, *N4bp3*, *Rab28* and *Tgm1*) showed no difference between hypertrophic and proliferative MCT cells. *P<0.05

To characterize the candidate *Col10a1* regulators identified by bioinformatics and proteomic approaches, we examined their correlation with *Col10a1* expression in MCT cells, which are mouse chondrocytes immortalized by SV40 large T antigen.^46^ MCT cells show properties of chondrocyte hypertrophy (increased level of Runx2 and other marker genes) and significantly upregulated *Col10a1* mRNA transcript (~10-fold) upon growth arrest (from 32 to 37 °C; Figure 5a). The results showed that most predictive candidate genes identified by MATCH program (*Foxj2*, *Klf3*/*Tef-2/*Cacd, *Mef2a*/RSRFC4 and *Nobox*/Og-2) were upregulated in hypertrophic MCT cells compared with proliferative MCT cells, whereas *Pou3f1*Tst-1 and Tbx5 showed no difference or was not detected in neither of the MCT cells (Figure 5b). Analysis of 10 of the candidate genes identified by Y1H showed that *Fn1*, *Hspa5*, *Psmb1*, *Ptgs2*/*cyclooxygenase 2* (*Cox-2*), *Rab1* and *Rps28* genes were significantly upregulated upon growth arrest (*P*<0.05), whereas genes *Anxa2*, *Nedd4*, *Rela* and *Rpl35* showed no difference (*P*>0.05, Figure 3c). Ten candidate genes identified by MS were analyzed. Only *Scrt2* and *Sfrp4* genes are significantly upregulated, whereas *Fus* gene is significantly downregulated in hypertrophic MCT cells, other genes showed no difference (Figure 5d).

### Analysis of candidate Col10a1 regulator Cox-2 (Ptgs2)

**Figure 6:**
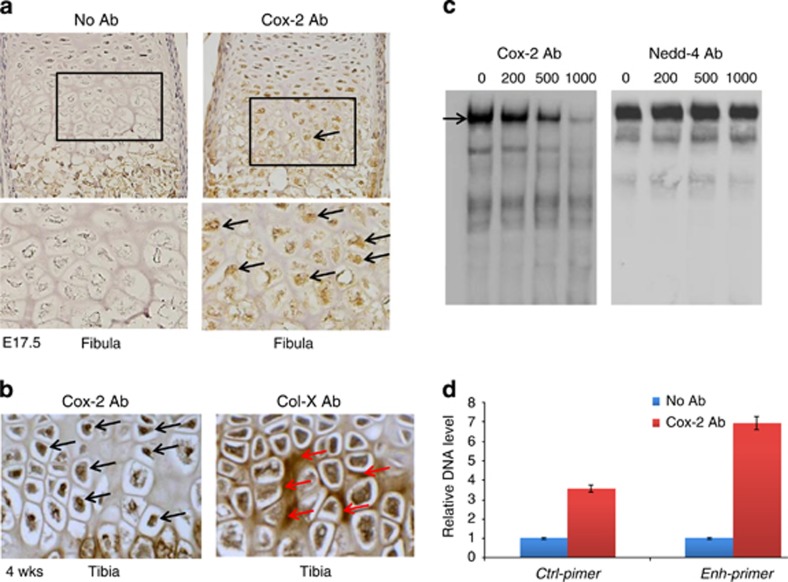
Cox-2 expression and interaction with *Col10a1* cis-enhancer. (**a**) IHC assay using Cox-2 antibody on sagittal sections of mouse limb (fibula) at E17.5 showed that Cox-2 is strongly expressed in nuclei of hypertrophic chondrocytes but not in resting or proliferative chondrocytes (black box and arrows). Bottom showed higher magnification of the boxed area. Left panel shows no antibody control. (**b**) IHC assay using Cox-2 antibody on tibia sections at 4 weeks' age also detected high-level Cox-2 expression in nuclei of hypertrophic chondrocytes (left panel, black arrows). Right panel shows IHC assay of collagen X, which is mostly expressed extracellular within hypertrophic zone (red arrows). (**c**) EMSA assay detected specific binding complex (black arrow) formed by the *Col10a1* enhancer and hypertrophic MCT cell nuclear extracts as previously described.^38^ The signal intensity decreased when 200 ng of Cox-2 antibody was added, 500 ng of antibody further reduced the signal, whereas 1000 ng of antibody only showed faint signal (left panel). No signal reduction was seen in parallel experiment in which gradient amount of Nedd4 antibody was used (right panel). Data of non-biotinylated competitor control were not shown. (**d**) Illustrated is the result of ChIP experiment showing precipitated DNA enriched by Cox-2 antibody or control IgG. qPCR using primers flanking the enhancer suggested a significant enrichment (~7-fold, *P*=0.034) of the enhancer by Cox-2 antibody, whereas sequence flanking the intronic control region did not show significant enrichment (~3-fold, *P*=0.062)

Cox-2 is a candidate *Col10a1*-interacting factor identified by Y1H screening and is significantly upregulated in hypertrophic MCT cells (Figures 3d and 5c). We chose to further analyze Cox-2 by immunohistochemical (IHC) analysis using *Cox-2* antibody and mouse embryonic (E17.5) long bone sections. The result showed that Cox-2 is strongly expressed in nuclei of hypertrophic chondrocytes but not in resting or proliferative chondrocytes (Figure 6a and data not shown). We also examined Cox-2 and collagen X expression post-natally (4 weeks' age). High-level Cox-2 expression was also observed within the nuclei of hypertrophic chondrocytes, whereas collagen X mostly expressed extracellular as expected (Figure 6b). We performed candidate electrophoretic mobility shift assay (EMSA) using the same DNA element form the *Col10a1* cis-enhancer and hypertrophic MCT cell nuclear extracts with gradient Cox-2 antibody.^38^ As an effort to characterize the few candidate genes (here in Nedd4) identified by Y1H as well as its associated N4bp3 (Nedd4-binding protein), we chose to perform similar candidate EMSA assay using Nedd4 antibody. The result supports that Cox-2, but not Nedd4, is a component of the DNA/protein complex (Figure 6c). We also performed chromatin immunoprecipitation (ChIP) assay using Cox-2 antibody (or IgG control) and the MCT cells. The result showed that the target sequence containing the *Col10a1* enhancer was significantly enriched (~7-fold, *P*=0.034) by Cox-2 antibody, whereas no significant enrichment was shown for the control primers (~3-fold, *P*=0.062; Figure 6d), suggesting an *in vivo* interaction between Cox-2 and the *Col10a1* cis-enhancer in MCT cells.

## Discussion

### Factors in addition to Runx2 are required for Col10a1 promote/enhancer activity

More than a decade ago, extensive studies focused on identification of the proximal promoter elements and their binding factors that can only mediate collagen X expression to certain level.^47, 48, 49, 50^ A highly conserved *Col10a1* distal promoter/enhancer element was later identified, which is able to mediate high-level hypertrophic chondrocyte-specific reporter expression in TG mice.^51^ We have recently narrowed down this enhancer to a 150-bp region.^37^ We have shown that a tandem-repeat Runx2 sites within its 3′-end is required for its enhancer activity *in vivo*, as mutating the Runx2 sites abolished its ability to drive cell-specific reporter expression.^38^ Notably, neither the 5′-sequence nor its overlapping 3′-sequence containing the Runx2 sites was able to drive cell-specific reporter expression, suggesting requirement of this entire 150-bp enhancer in mediating its enhancer activity. Interestingly, our detailed analysis revealed that the *Tg-4x150* mice show higher and more specific reporter expression than that of the *Tg-10kb* mice (Figure 1). This result confirmed the specificity of this 150-bp enhancer, whereas the 10-kb promoter/intronic elements contain nonspecific regulatory elements. The stronger reporter activity is most likely due to the multiple copies of the binding sites for important TFs (Runx2, AP1 etc.).^27,28,38^

### Bioinformatics prediction of TFBS for Col10a1 cis-enhancer

Identification of the 150-bp promoter as the genuine *Col10a1* enhancer makes it practical to predict potential TFBS that are functionally important.^52^ TFSEARCH is a pioneer web-based program that uses the TFMATRIX TFBS profile database to predict potential TFBSs.^53^ We identified several candidate factors including NF-kappaB, which has been shown to facilitate growth plate or longitudinal bone growth via BMP.^54^ The PROMO program was designed to search for known binding sites and TFs from the TRANSFAC database with updates.^55^ Among the 200 potential TFBSs, candidate factors AP1s, Hif and *α*MEF-2 were previously shown to regulate *Col10a1* expression and chondrocyte hypertrophy.^13,27,30,56,57^ Interestingly, the TRAP program identified approimately 50 TFs with a *P*-value <0.05, including factors that were selected by both the TFSEARCH and the PROMO programs (CdxA, Mef2 etc.). We only identified six potential TFs using the Match program (Figure 2d). We notice that the binding sites of TBX5 and CACD overlap with the tandem-repeat Runx2 sites, which we and others have previously characterized.^38,58^ Tbx5, which was not detected in hypertrophic MCT cells, has been shown to have a role in limb development.^59^ It is possible that the predictive candidate genes, especially those selected only by one program with a *P*-value >0.05, may be false-positive *Col10a1*-interacting factors. More attention should be paid to those factors identified by multiple web-based programs.

### Proteomic identification of novel interacting genes/factors

Multiple candidate factors were identified using the 150-bp enhancer as bait to screen the cDNA library derived from hypertrophic MCT cells. Not surprisingly, some candidate factors, including Rela (orp65), have been suggested to interact with this enhancer (Figure 2). Interestingly, we also identified *Ptgs2*/Cox-2, which was not selected by either of the above web-based programs. However, Cox-2 has been associated with chondrocyte hypertrophy and shows interaction with *Col10a1* cis-enhancer as described (Figure 6).^60^ Meanwhile, mass spectrometry analysis of the specific binding complex formed by the *Col10a1* enhancer and the MCT cell nuclear extracts identified multiple putative *Col10a1*-binding factors. Although some unrelated factors may be pulled down because of nonspecific force, we did identify Hnf-3, N4bp3 (Nedd4-binding protein) and other factors that were selected by *in silico* sequence analysis. Interestingly, we identified *Gdf7* with highest Mascot score and most matched peptides (Figures 4c and d,Supplementary Data S4). This is intriguing, as Gdf7 is a secreted protein, making it unlikely a gene expression regulator. However, Gdf7 have been associated with chondrocyte hypertrophy although the mechanism is not clear yet.^61^

### Identification of TFBS using comprehensive bioinformatics/proteomics approaches

Using web-based program to predict TFBS based on literature-derived data is an alternative and can significantly increase the efficiency of wet-lab experiments.^52^ Theoretically, the predictive candidate genes are expected to interact with the *Col10a1* enhancer and impact on *Col10a1* expression and chondrocyte hypertrophy. Indeed, MEF2C, which was identified by several programs, has been shown to control bone development by activating genes of chondrocyte hypertrophy, including *Col10a1*, whereas AP1/Ctgf interaction promotes chondrocyte hypertrophy during endochondral ossification.^50,56^ Interestingly, Pax family members were also identified by both PROMO (Pax-2, 4, 6, 8 and 9; Supplementary Data S2) and TRAP (Pax-2, *P*=0.0277 and Pax-7, *P*=0.0158; Supplementary Data S3) programs, whereas Pax-1 and Pax-9 have been linked to limb development with Pax-1 acting as a negative regulator for chondrocyte maturation.^62,63^ However, there are many predictive genes that show no evidence of correlation with the enhancer activity. These genes are either due to false selection of the program, or awaiting characterization before drawing any conclusion. Y1H approach is designed to screen for candidate genes interacting with cis-enhancer elements. In our studies, we were able to identify several genes (such as *Rela/p65*, *Ptgs2*) that are known to relate to chondrocyte hypertrophy during endochondral bone formation.^54,60^ Although Nedd4 was identified and may have a role in skeletal development, previous studies have suggested that Nedd4 is expressed in proliferating chondrocytes instead of hypertrophic chondrocytes.^64^ We did not detect difference of Nedd4 mRNA level in proliferative and hypertrophic MCT cells (Figure 5c), while candidate EMSA assay did not support that Nedd4 is a component of the specific binding complex (Figure 6c). Owing to leaky expression of reporter gene, false-positive clones may be selected. Similar situation may occur for candidate genes identified by mass spectrometry, considering their related (or irrelevant) expression and function.

We notice that MCT cells are transformed cells, which may not be a perfect cell model to represent hypertrophic differentiation and *Col10a1* upregulation and for the purpose of verification.^46^ However, we have performed expression analysis of most of the candidate genes in ATDC5 cells, another cell model of endochondral ossification, which also shows significant upregulation of *Col10a1* after extended culture and/or by ITS (insulin, transferrin and sodium selenite) induction.^65,66^ Similar levels of candidate gene expression was detected in ATDC5 cells compared with that in MCT cells, although with some discrepancy (data not shown). Further characterization of these candidate genes using both MCT and ATDC5 cell models will help to define their function in relation to *Col10a1* expression and chondrocyte hypertrophy *in vitro* (Figure 5).^46^

### Cox-2 and Col10a1 expression and chondrocyte hypertrophy

As mentioned above, Cox-2 was only selected by Y1H screening. This is intriguing, as Cox2 is a membrane-binding enzyme containing no known DNA-binding property. However, there are multiple lines of evidence which suggest the importance of Cox-2 upon bone formation. Cox-2 was shown to regulate mesenchymal cell differentiation and has essential roles in bone repair, whereas Cox-2 inhibitor negatively affect bone fracture healing.^67,68^ Cox-2 has been shown to control chondrocyte hypertrophy through cross-talk with BMP-2 pathway.^60^ We detected exclusive Cox-2 expression within nuclei of hypertrophic chondrocytes at both embryonic and postnatal stages. The involvement of Cox-2 within the specific binding complex, the increased Cox-2 expression upon *Col10a1* upregulation in hypertrophic MCT cells and the interaction between Cox-2 and *Col10a1* enhancer within MCT cells support a role of Cox-2 in *Col10a1* expression.

### Multiple factors in a putative Col10a1 regulatory mechanism

Along with others, we have demonstrated the indispensible role of Runx2 in regulation of its cell-specific expression across species.^36,38,69, 70, 71^ As to mouse type X collagen gene, multiple transcriptional regulators and signaling pathways have been shown to contribute to *Col10a1* expression. These include AP1 family members, PTH/PTHrP, SP3/SP1, GADD45*β*, MEF-2C, HIF-2a, SOX9, HDAC inhibitors, and the Ihh, Wnt or Bmp signaling pathways that have been reviewed recently.^2^ More importantly, most of the above factors/pathways show interaction with Runx2 and affect *Col10a1* expression and chondrocyte hypertrophy.^2^ In our current studies, by dissecting the *Col10a1* cis-enhancer, we identified multiple candidate *Col10a1*-interacting factors, including Cox-2 that has been shown to directly interact with Runx2.^72^ Indeed, candidate EMSA assays suggested that both Cox-2 and Runx2 are components of the specific binding complex (Figure 6).^38^ As Cox2 lacks DNA-binding site, suggesting that additional components exist in the binding complex. Given the direct interaction between Cox-2 and Runx2, we speculate that Cox-2 may act as a co-factor to interact with Runx2, which has DNA-binding capability, together to achieve their control over *Col10a1* expression and chondrocyte hypertrophy.^60,72^ There are also known repressors (such as Sox9) that have been associated with Runx2 together to regulate *Col10a1* expression, possibly by direct interaction, as both Runx2 and Sox9 sites are found adjacent each other within the *Col10a1* enhancer.^2,38,73^ In addition, some candidate repressors (such as Nfat) we identified show interaction with Runx2 and have a role in *Col10a1* expression and osteoarthritis development.^74,75^ Together, our data support that multiple factors, including transactivators, repressors and co-factors, work with Runx2 to regulate *Col10a1* expression and chondrocyte hypertrophy. We believe *Col10a1* trans-activators are promoters of chondrocyte hypertrophy and are targets for low bone growth as seen in skeletal dysplasia, whereas repressors will decrease *Col10a1* expression and delay chondrocyte hypertrophy and are, therefore, targets for bone over growth as seen in osteophyte formation in osteoarthritis. We also believe that the candidate *Col10a1* regulators identified by comprehensive biochemical, bioinformatics, proteomics and TG approaches as described in the article constitute unique resources. Further characterization of these candidate *Col10a1* regulators will open up new avenues of research that aims to better understanding skeletal developmental and disease mechanisms and may hold promise for pharmaceutical purpose to develop therapeutic targets for relevant skeletal diseases.

## Materials and Methods

### Histochemical analysis of Col10a1-reporter TG mice

*Tg* mice that use the large *Col10a1* promoter/intron and enhancer elements to drive *LacZ* gene as a reporter were generated as previously described.^37^ Specifically, the *Tg-10kb* TG construct contains a 6-kb *Col10a1* promoter and the large second intron (−6022 to +4220 bp) upstream of the *LacZ* gene. The *Tg-4x150bp* construct utilizes four copies of the 150-bp *Col10a1* enhancer (−4296 to −4147 bp) and the basal promoter (−220 to +110 bp) to drive the *LacZ* gene. Mouse embryos at the E15.5 (embryonic day 15.5) and P1 (postnatal day 1) stages from these two TG mouse lines were X-gal stained and compared for *LacZ* reporter expression. The limbs of these mouse embryos were paraffin embedded, sectioned and counterstained with nuclear fast red (Poly Scientific R&D Corp., Bay Shore, NY, USA). At least 30 sections of the growth plate and adjacent tissues were analyzed. Comparison was among littermates within same mouse line or between mouse lines at the same developmental stage (E15.5 or P1). The studies were approved by the animal care and oversight committee at Rush University Medical Center.

### *In silico* sequence analysis of Col10a1 cis-enhancer

The 150-bp *Col10a1* enhancer (−4296 to −4147 bp) was subjected to *in silico* sequence analysis to search for TFBS using following web-based programs: (1) TFSEARCH: the TFSEARCH tool was used to search for potential TFBS with a cutoff score of 85.^39,40^ The web address for TFSEARCH is at http://www.cbrc.jp/research/db/TFSEARCH.html. (2) Promo 3.0: Promo uses the TRANSFAC database (version 8.3) to identify TFBS in a given DNA sequence.^41,42^ The web address can be accessed at http://alggen.lsi.upc.es/cgi-bin/promo_v3/promo/promoinit.cgi?dirDB=TF_8.3. The searching result of potential TFBS will be displayed through a graphical interface or as a downloadable text file. (3) Transcription factor affinity prediction (TRAP): TRAP is used to predict which TFs are susceptible to bind a promoter with highest affinity.^43^ TRAP uses the TRANSFAC database and the searching result is listed in a table ranking the affinity of TFs with a *P*-value. TRAP is available at http://trap.molgen.mpg.de/cgi-bin/home.cgi. (4) MATCH: Match is a weight matrix-based program for predicting TFBS.^44,45^ With a courtesy of BIOBASE, we were able to use the TRANSFAC Professional database (version 10.1, January 2013). The address is at http://www.biobase-international.com/product/transcription-factor-binding-sites.

### Y1H screening

The 150-bp *Col10a1* enhancer was used as bait to screen for its binding factors from a cDNA library derived from hypertrophic MCT cells using the BD Matchmaker One-Hybrid System (Clontech, K1617-1) and the manufacturer suggested protocol with modifications. Specifically, the bait construct pHis2.1−2 × 150 was generated by cloning tandem copies of the 150-bp enhancer (2 × 150) into the MluI site of pHis2.1, a one-hybrid vector containing the nutritional reporter gene *HIS3* (Clontech, K1617-1). Detailed cloning strategy is available upon request. The cDNA library was generated using the BD SMART technology. The double strand (ds) cDNA derived from 1 *μ*g of total RNA of hypertrophic MCT cells was amplified by long-distance (LD)-PCR using vector-specific primers and was purified using the BD CHROMO SPIN Columns (Clontech, K1617-1). The Y187 yeast competent cells were prepared using manufacturer suggested protocol. Leaky expression of target-HIS3 reporter (pHis2.1−2 × 150) was suppressed by selective medium and 3-amino-1,2,4-triazole (3′-AT, Sigma, St. Louis, MO, USA). The GAL4 AD fusion library constructing and screening for one-hybrid interaction (target cis-element and its binding factors) was carried out by co-transformation of the BD SMART ds cDNA, the bait reporter construct pHis2.1−2 × 150, linearized pGADT7-Rec2 expression vector (PT3530-5, Clontech), and the pre-prepared Y187 yeast competent cells. Transformants that grow in selective medium (S.D./-His/-Leu/-Trp) and optimal 3-AT concentration (100 *μ*M) were harvested and subjected to PCR amplification using the Matchmaker Insert Check PCR Mix2 kit (cat. no. 630497, Clontech). PCR product was sequenced at the DNA Service Core at University of Illinois at Chicago using T7 primer or pGADT7-Rec2 expression vector-specific primers (Clontech). Sequence analysis and gene ID identification was carried out according to the National Center for Biotechnology Information (NCBI) database.

### Mass spectrometry analysis of DNA-binding complex

The DNA/protein complex formed by the *Col10a1* cis-enhancer (−4196 to −4172 bp) and the hypertrophic MCT cell nuclear extracts were analyzed by tandem mass spectrometry at the Proteomic and Biomarkers Core Facilities at Rush University Medical Center.^38^ The enhancer and its complementary oligo containing the tandem-repeat Runx2 sites (underlined: 5′-GATCCACAATTAGGTGTG GGTGTGGCCAGCA-3′) were synthesized with or without 5′-biotinylateion (IDT Technology, Coralville, IA, USA). The oligos were then annealed and incubated with 200 *μ*gs of MCT cell nuclear extracts for 1h at room temperature. This binding mixture was further incubated with streptavidin-conjugated magnetic particles (50 *μ*l, Invitrogen Corp., Carlsbad, CA, USA) to allow for efficient affinity pull-down of putative binding proteins. Proteins were eluted from the magnetic particles by addition of 40% methanol containing 0.1% formic acid. The protein elutes were then subjected to concentration via lyophilization and resuspended in 0.5 M guanidine HCl in PBS (pH 7.4). After digestion with sequencing grade trypsin (Promega Corp., Madison, WI, USA) for 24 h at 37 °C, the digested materials were spotted with 1 *μ*l of 1 mg/ml CHCA in 0.1% TFA and analyzed on an AXIMA QIT (Shimadzu Corp., Columbia, MD, USA) for protein identification. All runs were performed with technical replicates to ensure reproducibility of the data. Data analysis was carried out using the Mascot software platform (Matrix Science, London, UK). The SwissProt 51.6 (protein) database was searched with following parameters: Rodentia database, monoisotopic tryptic peptides only, peptide mass tolerance at ±2Da, fragment mass tolerance at ±1.5Da and maximum missed cleavages at 3. A decoy database was also searched to eliminate false-positive proteins. Confidence of protein identification was filtered to have a minimum MASCOT score of 35 and at least five matched peptides per protein identification.

### Candidate EMSAs

Candidate EMSA was performed using the LightShift Chemiluminescent EMSA kit (PIERCE, Rockford, IL, USA) with Cox-2 (H-62, sc-7951, Santa Cruz Biotechnology, Santa Cruz, CA, USA) and Nedd4 (A-16, sc-14429, Santa Cruz Biotechnology) antibodies. Shortly, the same *Col10a1* cis-enhancer and its complementary DNA oligos were annealed and incubated with 5 *μ*g of the MCT cell nuclear extracts with or without a series of diluted Cox-2 or Nedd4 antibody (at doses of 0, 0.2, 0.5 and 1 *μ*g, respectively) at room temperature for 20 min. The 5′-biotin-labeled and annealed oligos were then added to the binding mixture and further incubated for 30 min. The mismatched DNA sequence and non-biotin-labeled competitor control, the binding condition and the amount of probes used for EMSA were as previously described.^38^ After electrophoresis, the binding mixture within the gel was transferred to a positively charged Nylon membrane (Thermo Scientific, Rockford, IL, USA; cat. no. 77016). Detection of the biotin-labeled DNA using stabilized streptavidin-HRP conjugate and the chemiluminescent substrate module was performed according to manufacturer suggested protocol (PIERCE, cat. no. 89880). Visualization of the binding complex was to expose the membrane to X-ray film and the CCD camera of AlphaImager (Alpha Innotech, San Leandro, CA, USA).

### ChIP experiment

ChIP using MCT cells and Cox-2 antibody (sc-7951) was carried out using the Pierce Agarose ChIP Kit (cat. no. 26156, Thermo Scientific) as described.^38^ Briefly, sub-confluent MCT cells grown at 32 °C were further incubated at 37 °C for additional 3 days. These cells were then cross-linked by 1% formaldehyde for 10 min at room temperature before addition of glycine to quench cross-linking. After harvest, cells were treated with S1 nuclease to shear chromatin DNA to majorly 200–400 bp. Immunoprecipitation was conducted using 10% of total pre-cleared chromatin (input sample) and 5 *μ*g each of the Cox-2 antibody or control IgG. The cross-linked and precipitated protein mixtures were then subjected to proteinase K digestion and reverse cross-linking to allow for DNA purification. Semiquantitative and real-time PCR using DNA elutes and specific primers flanking the *Col10a1* cis-enhancer (forward: 5′-CTGAACAGCTCCGAGGAAAC-3′, reverse: 5′-TGGATATTCAGCCCTTTTGG-3′) and associated intron II (forward: 5′-AATGATGCATGGAAACGACA-3′, reverse: 5′-GCCTATGCAATTGTTTTTAGCTT-3′) were performed, as described.^38^ Data of real-time PCR is collected from multiple runs with duplicate templates. *P*<0.05 implies significant enrichment of target sequence using Cox-2 antibody.

### Cell culture, RNA extraction and qRT-PCR

MCT cells were cultured at 32 °C in standard DME media with 8% fetal bovine serum and 8% CO_2_ as described.^46^ MCT cells grown at 32 °C and reached 70–80% confluence were further cultured at 37 °C for additional 3 days to obtain hypertrophic property. These cells were harvested for total RNA preparation using Trizol (Invitrogen Corp.). These total RNAs were subjected to first-strand cDNA synthesisis using Superscript III reverse transcriptase (Invitrogen Corp.). Quantitative real-time PCR was performed using the MyiQ Single Color Real-Time PCR Detection System and SYBR Green master mix (Bio-Rad, Hercules, CA, USA). Gene-specific primers for *Gapdh*, *Col10a*, *Runx2*, as well as candidate *Col10a1* regulators were listed in primer tables 1, 2 and 3 respectively (Supplementary Data S6). *Gapdh* was used as an internal control of RNA quality. *Col10a1* and *Runx2* were analyzed to confirm the upregulation of *Col10a1* and the hypertrophic property of MCT cells. The mean CT (threshold cycle number) values indicating relative transcript levels of target genes were normalized to *Gapdh* and were analyzed using 2^−ΔΔ^Ct method and student *t-*test.^36,47^ Data are collected from multiple runs with duplicate templates. *P*<0.05 implies significant fold change of target genes in hypertrophic MCT cells.

### IHC analysis

Sagittal sections of mouse hind limbs at the age of E17.5 and 4 weeks were subjected to IHC analysis using Cox-2 (H-62, sc-7951, Santa Cruz) and collagen X (E-14, sc-323750, Santa Cruz) antibodies. Briefly, paraffin-embedded limb sections undergone de-paraffin and rehydration were subjected to antigen retrieval by incubation with hot (95 °C) sodium citrate buffer (0.01 M, pH 6.0) for 10 min. The tissue sections were then exposed to hydrogen peroxide (3% H_2_O_2_) for 5 min to quench the endogenous peroxidase, followed by blocking with 30% horse serum (30 min). The slides were incubated overnight with above primary Cox-2 or collagen X antibodies (1 : 50 dilution) at 4 °C. Non-immune mouse IgG was used as a negative control. After washing with the 1xTBST (Tris Buffered Saline with 0.1% Tween-20), the slides were further incubated with biotinylated secondary antibody (anti-rabbit IgG, Santa Cruz) and detected using the ABC kit (Elite PK-6200 Universal, VECTOR Laboratories, Burlingame, CA, USA). Slides were counterstained with nuclear fast red (Poly Scientific R&D Corp.) and analyzed using Nikon Eclipse 80i, (Nikon Instruments Inc., Melville, NY, USA) and the Qcapture Suite software (version, 2.95.0, Quantitative Imaging Corp., Surrey, BC, Canada).

## Abbreviations

SMCD: Schmid metaphyseal chondrodysplasia
CCD: cleidocranial dysplasia
TF: transcription factor
TRAP: transcription factor affinity prediction
MCT cells: mouse chondrocytes immortalized with large T antigen
Cox-2: cyclooxygenase 2
Y1H: yeast one-hybrid screening
ChIP: chromatin immunoprecipitation
TFBS: transcription factor binding sites
EMSA: electrophoretic mobility shift assay
IHC: immunohistochemistry
TG: Transgenic
