# Supplementary material for: Identification and characterization of the novel Col10a1 regulatory mechanism during chondrocyte hypertrophic differentiation
Source: Cell Death Dis. 2014 Oct 16;5(10):e1469–. doi: 10.1038/cddis.2014.444 (PMC4649528; doi:10.1038/cddis.2014.444)
Supplement: Supplementary Information [file cddis2014444x1.pdf]

TFSEARCH Search Result

```
** TFSEARCH  ver.1.3 **   (c)1995 Yutaka Akiyama (Kyoto Univ.)

 This simple routine searches highly correlated sequence fragments
 versus TFMATRIX transcription factor binding site profile database
 by E.Wingender, R.Knueppel, P.Dietze, H.Karas (GBF-Braunschweig).

  <Warning> Scoring scheme is so straightforward in this version.
            score = 100.0 * ('weighted sum' - min) / (max - min)
            The score does not properly reflect statistical significance!

 Database:  TRANSFAC MATRIX TABLE, Rel.3.3 06-01-1998
 Query:     M150bpTFSearch (150 bases)
 Taxonomy:  Vertebrate
 Threshold: 85.0 point


 TFMATRIX entries with High-scoring:

   1 GCCTCCTGTT TCACGTAGAA TAAGCTCCTT CATAAAGTCA CAGACCAGTC entry        score
                                      <------               M00100 CdxA   96.2
                                        <--------            M00041 CRE-BP 87.2
                                      <------               M00101 CdxA   87.1
                     <-------                               M00101 CdxA   85.7

  51 AGGCTGAACA GCTCCGAGGA AACACCCAGA ATAAAAATAG TTTAATACAC entry        score
                                                 ------>    M00101 CdxA   92.9
                       <----------                          M00053 c-Rel  91.7
                                                      ----- M00099 S8     90.4
                         ------->                           M00148 SRY    89.1
                       <----------                          M00052 NF-kap 88.1
                                        ------>             M00101 CdxA   85.7
                       <----------                          M00054 NF-kap 85.4
                                 <-------                   M00101 CdxA   85.0

 101 ACAATTAGGT GTGGGTGTGG CCAGCAAATA CTCTGATTCT ACAATCTGTT entry        score
      <-------                                              M00241 Nkx-2. 91.2
     ----------->                                           M00099 S8     90.4
        <-----------                                        M00073 deltaE 87.4
                            <------------                   M00131 HNF-3b 86.7


 Total 15 high-scoring sites found.
 Max score: 96.2 point, Min score: 85.0 point
```

- TRANSFAC database document (Notice to users)
- TFMATRIX: binding site distribution matrix

- **Parallel Application Tsukuba Laboratory** Home Page

- **PAPIA system** Top Page

[Save link (only for 7 days): http://alggen.lsi.upc.es/cgi-bin/promo_v3/promo/promo.cgi?dirDB=TF_8.3&idCon=137124360600&getFile=resumSearchRes.html]

Factors predicted within a dissimilarity margin less or equal than 15 % :

0 p300 [T01427]	1 FACB [T02841]	2 BTEB3 [T05051]	3 FOXD3 [T02290]	4 PR B [T00696]	5 PR A [T01661]	6 Pu box binding factor [T00704]	7 GR-alpha [T00337]
8 TRM1 [T05311]	9 HNF-3beta [T01049]	10 Msx-1 [T02072]	11 HBP-1a(1) [T01394]	12 HBP-1a(c14) [T01395]	13 TGA1a [T00829]	14 USF [T00873]	15 HES-1 [T01649]
16 HTF [T05026]	17 POU1F1a [T00691]	18 C/EBPdelta [T00109]	19 unc-86 [T01882]	20 GA-BF [T00297]	21 BR-C Z2 [T01478]	22 Ncx [T04368]	23 Cdx-1 [T01484]
24 PHR1 [T05369]	25 HOXD8 [T01426]	26 HOXD8 [T01754]	27 Gt [T00328]	28 R2 [T00712]	29 HNF-3 [T02277]	30 POU1F1b [T01516]	31 POU1F1c [T01902]
32 HNF-3beta [T02513]	33 AP-1 [T00029]	34 AP-1 [T00032]	35 DEF:GLO:SQUA [T03217]	36 USF2 [T02115]	37 c-Fos [T00123]	38 Zta [T00923]	39 Pax-2 [T01823]
40 HOXA3 [T00378]	41 TFIIB [T00818]	42 Pax-2a [T00678]	43 Pax-6 [T00682]	44 RC2 [T00724]	45 MF3 [T00507]	46 Zic3 [T04671]	47 NHP-1 [T00621]
48 NF-1 [T01298]	49 AP-2alphaA [T00035]	50 NF-1 (-like proteins) [T00601]	51 AR [T00040]	52 GR [T00333]	53 HNF-3beta [T02344]	54 MYB2 [T02536]	55 CREMtau [T01309]
56 CREMtau1 [T02108]	57 CREMtau2 [T02109]	58 myogenin [T00528]	59 MyoD [T00526]	60 Nkx2-1 [T00857]	61 PDR3 [T03525]	62 STAT5A [T04683]	63 IRF-1 [T00423]
64 PEA3 [T00684]	65 HELIOS [T06012]	66 Elk-1 [T00250]	67 c-Ets-2 [T00113]	68 STE12 [T00772]	69 IRF-3 [T04673]	70 PU.1 [T02068]	71 HNF-3alpha [T00371]
72 NF-AT1 [T00550]	73 ABI4 [T05743]	74 Pax-9a [T03593]	75 Pax-9b [T03594]	76 Nrf2:MafK [T05666]	77 LVb-binding factor [T00477]	78 MBF1 [T00492]	79 Croc [T02291]
80 POU3F1 [T00969]	81 Cutl1 [T02042]	82 AGL3 [T03025]	83 HOXD9 [T01424]	84 HOXD10 [T01425]	85 HOXD9 [T01755]	86 HOXD9 [T01756]	87 HOXD10 [T01757]
88 HOXD10 [T01758]	89 Nkx6-2 [T02050]	90 FOXF1 [T02461]	91 HNF-3alpha [T02512]	92 Pax-4a [T02983]	93 POU3F2 [T00630]	94 MEF-2DAB [T02505]	95 FOXP3 [T04280]
96 SPF1 [T03975]	97 aMEF-2 [T01006]	98 SBF-1 [T00739]	99 Tll [T00789]	100 Antp [T00026]	101 Prd [T00699]	102 MRF-2 [T04675]	103 C/EBPalpha [T00107]
104 Sox13 [T02420]	105 SRY [T00997]	106 HNF-1B [T01950]	107 S8 [T01483]	108 Ubx [T00863]	109 Crx [T03461]	110 Cart-1 [T03978]	111 DPBF-1 [T04363]
112 DPBF-2 [T04364]	113 Xvent-2 [T04663]	114 HOXA4 [T00128]	115 Zic1 [T04669]	116 Zic2 [T04670]	117 Isl-1 [T01956]	118 USF-1 [T00875]	119 WT1 I [T01840]
120 Spz1 [T04668]	121 VDR [T00885]	122 NF-1 [T00537]	123 ENKTF-1 [T00255]	124 C/EBPbeta [T00017]	125 E12 [T01786]	126 GT-1 [T00339]	127 DBP [T00183]
128 Pax-8 [T01828]	129 HSF1 (long) [T01042]	130 HSF1 (short) [T02104]	131 C/EBPbeta(p20) [T01420]	132 DSXF [T00955]	133 DSXM [T00956]	134 NF-X3 [T01514]	135 En-1 [T02016]
136 CUTL1 [T00100]	137 Sox2 [T01836]	138 C/EBPgamma [T02028]	139 ABF1 [T00056]	140 TCF-1(P) [T01109]	141 TCF-3 [T02857]	142 Xvent-1 [T04665]	143 C/EBPalpha [T00104]
144 HNF-3gamma [T01050]	145 POU2F2 (Oct-2.1) [T00646]	146 MAC1 [T01265]	147 C/EBPalpha [T00105]	148 C/EBPalpha [T00108]	149 C/EBPbeta [T00459]	150 C/EBPbeta [T00581]	151 E47 [T00207]
152 CP2 [T00152]	153 Alfin1 [T04733]	154 Vpr [T02399]	155 RF2a [T02811]	156 Egr-3 [T00243]	157 Egr-3 [T05889]	158 AP-3 (2) [T00039]	159 MCM1 [T00500]
160 Zen-1 [T00917]	161 Zen-2 [T02099]	162 Eve [T00272]	163 BR-C Z3 [T01479]	164 TBP [T00798]	165 NF-kappaB [T00590]	166 NF-AT2 [T01945]	167 NF-AT1 [T01948]
168 NF-AT1 [T01944]	169 HMG I(Y) [T02368]	170 STAT4 [T01577]	171 c-Ets-1 [T00112]	172 FOXN2 [T04206]	173 STAT1beta [T01573]	174 PU.1 [T00702]	175 PDR1 [T03524]
176 GAL4 [T00302]	177 PR B [T00697]	178 GR-beta [T01920]	179 USF2b [T02377]	180 c-Jun [T00131]	181 JunB [T00436]	182 c-Fos [T00124]	183 c-Jun [T00132]
184 c-Jun [T00133]	185 JunD [T00437]	186 c-Fos [T00122]	187 ATF3 [T01095]	188 CREMalpha [T01803]	189 LCR-F1 [T01599]	190 POU2F1 [T01466]	191 FOXO3a [T02938]
192 Pax-6 [T01122]	193 RITA-1 [T02786]	194 Opaque-2 [T00668]					

Zoom Data (txt)

1	10	20	30	40	50	60	70	80	90	100	110	120	130	140	150	160
MColX_150	0 1 2 3<br>4 5 6 7<br>8 9 11 12<br>14 29 40 49<br>53 54 60 65<br>66 68 71 76<br>192 193 194	10 13 15 16<br>17 18 19 20<br>21 24 25 26<br>27 129 130 132<br>133 191	0 1 22 23<br>28 65 66 164<br>189 190	10 14 22 23<br>25 26 29 30<br>31 32 33 34<br>35 36 37 38<br>39 40 41 42<br>43 47 179 180<br>181 182 183 184<br>185 186 187 188	33 34 36 37<br>39 43 44 45<br>46 47 48 49<br>50 58 115 116<br>151 152 179 180<br>181 182 183 184<br>185 186 187	4 5 7 40<br>51 52 53 54<br>55 56 57 58<br>59 61 176 177<br>178	0 1 3 4<br>5 6 7 8<br>9 29 53 60<br>61 62 63 64<br>65 66 67 68<br>69 70 71 72<br>146 165 166 167<br>168 169 170 171<br>172 173 174 175	10 17 18 19<br>20 21 40 44<br>45 46 48 54<br>73 74 75 76<br>77 78 79 83<br>84 85 86 87<br>88 90 115 116<br>120 121 129 130<br>151 152 164	17 18 19 21<br>22 23 25 26<br>29 30 31 32<br>54 68 80 81<br>82 89 91 92<br>93 94 95 96<br>97 98 145 163	10 17 18 19<br>21 22 23 38<br>40 81 99 100<br>101 102 104 105<br>106 108 109 111<br>112 113 119 136<br>137 159 160 161<br>162	10 17 18 19<br>21 22 23 40<br>44 46 73 74<br>75 81 99 100<br>101 103 107 108<br>109 110 114 115<br>116 117 118 119<br>120 135 154 156<br>157 158	10 38 40 44<br>45 46 48 55<br>56 57 73 74<br>75 115 116 118<br>119 121 122 123<br>124 125 151 152<br>153 154 155	17 18 19 21<br>29 58 59 76<br>80 81 82 89<br>91 92 93 102<br>103 126 127 128<br>140 141 142 143<br>144 145 146 147<br>148 149 150	17 18 19 20<br>21 40 81 104<br>105 129 130 131<br>132 133 136 137<br>138 139	17 18 19 21<br>40 54 103 134<br>135	

Distribution of the nucleotides over the given chain:

A	32.7%
C	23.3%
G	19.3%
T	24.7%

Transcription factor Affinity Prediction (TRAP) Web Tools

Home TRAP PASTAA Gbrowse Help Authors Download Cite

TRAP (single sequence)

calculating... please wait...the results will appear below

Sequence: **MColX_150**

∗ The table is ranked from the lowest to the highest p-value.

Results are displayed in the table below **[save table].**

Affinity-based ranking of transcription factors

Rank	P-value	Corrected p-value	Matrix ID	Matrix name	Affinity Plot	Binding Sites
1	0.00153	0.737	M00395	V$HOXA3_01	Graph	Sites
2	0.00317	0.737	M01440	V$LHX8_01	Graph	Sites
3	0.00327	0.737	M01331	V$ISX_01	Graph	Sites
4	0.00406	0.737	M01421	V$LHX4_01	Graph	Sites
5	0.00526	0.737	M00403	V$AMEF2_Q6	Graph	Sites
6	0.00646	0.737	M00150	V$BRACH_01	Graph	Sites
7	0.00675	0.737	M01386	V$EVX2_01	Graph	Sites
8	0.0083	0.737	M01371	V$GBX1_01	Graph	Sites
9	0.00981	0.737	M01113	V$CACD_01	Graph	Sites
10	0.0142	0.737	M01162	V$OG2_01	Graph	Sites
11	0.0144	0.737	M00640	V$HOXA4_Q2	Graph	Sites
12	0.0158	0.737	M01339	V$PAX7_01	Graph	Sites
13	0.0175	0.737	M01388	V$DLX5_01	Graph	Sites
14	0.018	0.737	M00415	V$AREB6_04	Graph	Sites
15	0.019	0.737	M00423	V$FOXJ2_02	Graph	Sites
16	0.0203	0.737	M00133	V$TST1_01	Graph	Sites
17	0.0206	0.737	M01019	V$TBX5_01	Graph	Sites
18	0.0213	0.737	M01020	V$TBX5_02	Graph	Sites
19	0.0236	0.737	M00100	V$CDXA_01	Graph	Sites
20	0.0236	0.737	M00935	V$NFAT_Q4_01	Graph	Sites
21	0.0249	0.737	M01037	V$GLI_Q2	Graph	Sites
22	0.0257	0.737	M00821	V$NRF2_Q4	Graph	Sites
23	0.0265	0.737	M00053	V$CREL_01	Graph	Sites
24	0.0272	0.737	M00099	V$S8_01	Graph	Sites
25	0.0274	0.737	M00250	V$GFI1_01	Graph	Sites
26	0.0277	0.737	M00486	V$PAX2_02	Graph	Sites
27	0.028	0.737	M00231	V$MEF2_02	Graph	Sites
28	0.0329	0.737	M01431	V$BARX2_01	Graph	Sites
29	0.0329	0.737	M01301	V$MEF2_05	Graph	Sites

30	0.0348	0.737	M01023	V$HSF1_Q6	Graph	Sites	
31	0.0357	0.737	M00246	V$EGR2_01	Graph	Sites	
32	0.0365	0.737	M00407	V$RSRFC4_Q2	Graph	Sites	
33	0.037	0.737	M01462	V$POU6F1_02	Graph	Sites	
34	0.0374	0.737	M01044	V$TBX5_Q5	Graph	Sites	
35	0.0382	0.737	M01349	V$HB9_01	Graph	Sites	
36	0.0386	0.737	M01446	V$BARHL2_01	Graph	Sites	
37	0.0387	0.737	M00026	V$RSRFC4_01	Graph	Sites	
38	0.0388	0.737	M00405	V$MMEF2_Q6	Graph	Sites	
39	0.0403	0.737	M01588	V$GKLF_02	Graph	Sites	
40	0.041	0.737	M01657	V$GLI3_Q5_01	Graph	Sites	
41	0.0415	0.737	M01292	V$HOXA13_01	Graph	Sites	
42	0.0426	0.737	M01422	V$LHX61_02	Graph	Sites	
43	0.044	0.737	M00991	V$CDX_Q5	Graph	Sites	
44	0.0452	0.737	M01332	V$BARHL1_01	Graph	Sites	
45	0.046	0.737	M01016	V$SOX17_01	Graph	Sites	
46	0.0477	0.737	M01436	V$CRX_02	Graph	Sites	
47	0.0481	0.737	M00292	V$FREAC4_01	Graph	Sites	
48	0.0489	0.737	M01212	V$STAT1STAT1_Q3	Graph	Sites	
49	0.0509	0.737	M00641	V$HSF_Q6	Graph	Sites	
50	0.0518	0.737	M01429	V$HOMEZ_01	Graph	Sites	
51	0.0556	0.737	M01239	V$RELBP52_01	Graph	Sites	
52	0.0559	0.737	M01365	V$EN1_02	Graph	Sites	
53	0.0566	0.737	M00774	V$NFKB_Q6_01	Graph	Sites	
54	0.0569	0.737	M00052	V$NFKAPPAB65_01	Graph	Sites	
55	0.0589	0.737	M01455	V$EN2_01	Graph	Sites	
56	0.061	0.737	M00105	V$CDPCR3_01	Graph	Sites	
57	0.0613	0.737	M01399	V$HB24_01	Graph	Sites	
58	0.0614	0.737	M01325	V$LH2_01	Graph	Sites	
59	0.063	0.737	M01403	V$OTX3_01	Graph	Sites	
60	0.063	0.737	M01448	V$HOXD1_01	Graph	Sites	
61	0.0631	0.737	M01382	V$GBX2_01	Graph	Sites	
62	0.0631	0.737	M01355	V$ALX3_01	Graph	Sites	
63	0.0633	0.737	M00081	V$EVI1_04	Graph	Sites	
64	0.0639	0.737	M01384	V$RHOX11_02	Graph	Sites	
65	0.0641	0.737	M01330	V$HOXB3_01	Graph	Sites	
66	0.0653	0.737	M00251	V$XBP1_01	Graph	Sites	
67	0.0659	0.737	M00420	V$MEIS1AHOXA9_01	Graph	Sites	
[illegible]	[illegible]	[illegible]	[illegible]	[illegible]	[illegible]	[illegible]	

search tools logout help

Result what is this?

Return to Match

Match results filename : Save Download

Matrix library : TRANSFAC MATRIX TABLE, Release 2013.1

Sequence file : 150.seq

Profile : vertebrate_non_redundant_minFP.prf

Only high-quality matrices : YES

Cut-offs : Minimize false positives

Total sequence length : 150

Total sites number : 6

Frequency of sites : 0.040000

manage your data:

Sequences: uploaded sequences are automatically stored to your user account. You may delete sequences that you no longer need here.

Matrices: the complete TRANSFAC matrix library is available for use with Match, but you may also choose to Create or edit your own matrices. You may additionally Compare your matrices against the TRANSFAC matrix library

Profiles: a selection of commonly used profiles (collection of matrices) is made available for use with Match. Alternatively you may choose to Create or edit your own profile, selecting from the matrices within the TRANSFAC matrix library as well as any custom matrices that you have created.

Saved Match results: each time a Match analysis is run you are given the option to save the results to your user account. You may view and manage your saved results here.

Scanning sequence ID: 150.seq_0 - View a graphical output of the following search result

Show 10 entries

Matrix Id	Position(Strand)	Core Score	Matrix Score	Sequence	Factor Name
V$TST1_01	76 (+)	0.929	0.903	ccaGAATAaaaatag	Tst-1
V$RSRFC4_Q2	78 (+)	1	0.905	agaataaaAATAGttta	RSRFC4
V$FOXJ2_02	81 (+)	0.897	0.905	ataAAAATagttta	FOXJ2
V$OG2_01	102 (-)	1	1	cAATTA	OG-2
V$TBX5_01	105 (+)	1	0.972	ttaGGTGTgggt	TBX5
V$CACD_01	113 (-)	1	1	GGGTGtgg	CACD

Mascot Search Results

```
User                    : Praneeth Edirisinghe
Email                   :
Search title            : Ced 2
MS data file            : Ced_new2.mgf
Database                : SwissProt 51.6 (257964 sequences; 93947433 residues)
Taxonomy                : Rodentia (Rodents) (19706 sequences)
Timestamp               : 10 Mar 2011 at 21:00:36 GMT
Enzyme                  : Trypsin
Mass values             : Monoisotopic
Protein Mass            : Unrestricted
Peptide Mass Tolerance  : ± 2 Da
Fragment Mass Tolerance : ± 1.5 Da
Max Missed Cleavages    : 3
Instrument type         : Default
Number of queries       : 9192
Protein hits            : GDF7_MOUSE   Growth/differentiation factor 7 precursor (GDF-7) - Mus musculus (Mouse)
                          ALBU_MOUSE   Serum albumin precursor - Mus musculus (Mouse)
                          K2C1_MOUSE   Keratin, type II cytoskeletal 1 (Cytokeratin-1) (CK-1) (Keratin-1) (K1)
                          RALY_MOUSE   RNA-binding protein Raly (hnRNP associated with lethal yellow protein) (
                          HRBL_MOUSE   HIV-1 Rev-binding protein-like protein (Rev/Rex activation domain-bindin
                          ODPAT_RAT    Pyruvate dehydrogenase E1 component alpha subunit, testis-specific form,
                          ODPAT_MOUSE  Pyruvate dehydrogenase E1 component alpha subunit, testis-specific form,
                          FUS_MOUSE    RNA-binding protein FUS (Pigpen protein) - Mus musculus (Mouse)
                          SCN3A_RAT    Sodium channel protein type 3 subunit alpha (Sodium channel protein type
                          THNSL_MOUSE  Threonine synthase-like 1 - Mus musculus (Mouse)
                          SCRT2_MOUSE  Transcriptional repressor scratch 2 (Scratch homolog 2 zinc finger prote
                          ADR1_MOUSE   Adiponectin receptor protein 1 - Mus musculus (Mouse)
                          NDUV2_MOUSE  NADH dehydrogenase [ubiquinone] flavoprotein 2, mitochondrial precursor
                          PTN21_RAT    Tyrosine-protein phosphatase non-receptor type 21 (EC 3.1.3.48) (Protein
                          DC1L1_RAT    Cytoplasmic dynein 1 light intermediate chain 1 (Dynein light intermedia
                          PHX2A_MOUSE  Paired mesoderm homeobox protein 2A (Paired-like homeobox 2A) (PHOX2A ho
                          KT3K_MOUSE   Ketosamine-3-kinase (EC 2.7.1.-) (Fructosamine-3-kinase-related protein)
                          CONA1_MOUSE  Collagen alpha-1(XXIII) chain - Mus musculus (Mouse)
                          NFH_RAT      Neurofilament triplet H protein (200 kDa neurofilament protein) (Neurofi
                          NEUG_MOUSE   Neurogranin (Ng) (RC3) - Mus musculus (Mouse)
                          SEM5A_MOUSE  Semaphorin-5A precursor (Semaphorin F) (Sema F) - Mus musculus (Mouse)
                          CD2L5_MOUSE  Cell division cycle 2-like protein kinase 5 (EC 2.7.11.22) (CDC2-related
                          CAC1H_RAT    Voltage-dependent T-type calcium channel subunit alpha-1H (Voltage-gated
                          MPIP1_RAT    M-phase inducer phosphatase 1 (EC 3.1.3.48) (Dual specificity phosphatas
                          RL14_MOUSE   60S ribosomal protein L14 - Mus musculus (Mouse)
                          CAC1H_MOUSE  Voltage-dependent T-type calcium channel subunit alpha-1H (Voltage-gated
                          DPOD1_RAT    DNA polymerase delta catalytic subunit (EC 2.7.7.7) - Rattus norvegicus
                          BOREA_MOUSE  Borealin (Cell division cycle-associated protein 8) (MESrg) - Mus muscul
                          CBX6_MOUSE   Chromobox protein homolog 6 - Mus musculus (Mouse)
                          DOCK9_MOUSE  Dedicator of cytokinesis protein 9 (Cdc42 guanine nucleotide exchange fa
                          IL33_RAT     Interleukin-33 precursor (IL-33) - Rattus norvegicus (Rat)
                          RM36_MOUSE   39S ribosomal protein L36, mitochondrial precursor (L36mt) (MRP-L36) - M
                          COBA1_MOUSE  Collagen alpha-1(XI) chain precursor - Mus musculus (Mouse)
                          EP15_MOUSE   Epidermal growth factor receptor substrate 15 (Protein Eps15) (AF-1p pro
                          PRP8_MOUSE   Pre-mRNA-processing-splicing factor 8 (Splicing factor Prp8) - Mus muscu
                          ALBU_RAT     Serum albumin precursor - Rattus norvegicus (Rat)
                          STAB1_MOUSE  Stabilin-1 precursor (FEEL-1 protein) - Mus musculus (Mouse)
                          HNF3A_MOUSE  Hepatocyte nuclear factor 3-alpha (HNF-3A) (Forkhead box protein A1) - M
                          TUB_MOUSE    Tubby protein - Mus musculus (Mouse)
                          TUB_RAT      Tubby protein homolog - Rattus norvegicus (Rat)
                          K0515_MOUSE  Uncharacterized protein KIAA0515 - Mus musculus (Mouse)
                          EPS8_MOUSE   Epidermal growth factor receptor kinase substrate 8 - Mus musculus (Mous
                          BAT2_RAT     Large proline-rich protein BAT2 (HLA-B-associated transcript 2) - Rattus
                          FZD8_MOUSE   Frizzled-8 precursor (Fz-8) (mFz8) - Mus musculus (Mouse)
                          FGRL1_MOUSE  Fibroblast growth factor receptor-like 1 precursor (FGF receptor-like pr
                          HRSL3_MOUSE  HRAS-like suppressor 3 (H-rev 107 protein) - Mus musculus (Mouse)
                          COBA2_MOUSE  Collagen alpha-2(XI) chain precursor - Mus musculus (Mouse)
                          3BHS1_MOUSE  3 beta-hydroxysteroid dehydrogenase/Delta 5-->4-isomerase type I (3-beta
```


Created with novaPDF Printer (www.novaPDF.com). Please register to remove this message.

3BHS6_MOUSE	3 beta-hydroxysteroid dehydrogenase/Delta 5-->4-isomerase type VI (3-beta
3BHS2_MOUSE	3 beta-hydroxysteroid dehydrogenase/Delta 5-->4-isomerase type II (3-beta
CC45L_MOUSE	CDC45-related protein (PORC-PI-1) - Mus musculus (Mouse)
RAIN_MOUSE	Ras-interacting protein 1 (Rain) - Mus musculus (Mouse)
SFRP4_MOUSE	Secreted frizzled-related sequence protein 4 precursor (sFRP-4) (Frizzle
PRAM_MOUSE	PML-RARA-regulated adapter molecule 1 (PRAM-1) - Mus musculus (Mouse)
AKA11_RAT	A-kinase anchor protein 11 (Protein kinase A-anchoring protein 11) (PRKA
GPR27_MOUSE	Probable G-protein coupled receptor 27 (Super conserved receptor express
FOXK2_MOUSE	Forkhead box protein K2 (Interleukin enhancer-binding factor 1) (Cellula
RB_RAT	Retinoblastoma-associated protein (PP105) (RB) (Fragment) - Rattus norve
LPHN1_RAT	Latrophilin-1 precursor (Calcium-independent alpha-latrotoxin receptor 1
SUCA_MOUSE	Succinyl-CoA ligase [GDP-forming] subunit alpha, mitochondrial precursor
ARMX2_MOUSE	Armadillo repeat-containing X-linked protein 2 - Mus musculus (Mouse)
DDEF2_MOUSE	Development and differentiation-enhancing factor 2 (Pyk2 C-terminus-asso
NFH_MOUSE	Neurofilament triplet H protein (200 kDa neurofilament protein) (Neurofi
FCN1_RAT	Ficolin-1 precursor (Ficolin-A) (Ficolin-alpha) (M-ficolin) (Collagen/fi
MYG_RAT	Myoglobin - Rattus norvegicus (Rat)
LRC8E_MOUSE	Leucine-rich repeat-containing protein 8E - Mus musculus (Mouse)
GPR27_RAT	Probable G-protein coupled receptor 27 (Super conserved receptor express
RM12_MOUSE	39S ribosomal protein L12, mitochondrial precursor (L12mt) (MRP-L12) - M
ADH1_RAT	Alcohol dehydrogenase 1 (EC 1.1.1.1) (Alcohol dehydrogenase A subunit) -
LTBP3_MOUSE	Latent-transforming growth factor beta-binding protein 3 precursor (LTBP
AT10D_MOUSE	Probable phospholipid-transporting ATPase VD (EC 3.6.3.1) (ATPVD) - Mus
N4BP3_MOUSE	NEDD4-binding protein 3 (N4BP3) - Mus musculus (Mouse)
CUL7_MOUSE	Cullin-7 (CUL-7) (p185) (p193) - Mus musculus (Mouse)
CHMP6_MOUSE	Charged multivesicular body protein 6 (Chromatin-modifying protein 6) -
MAST2_MOUSE	Microtubule-associated serine/threonine-protein kinase 2 (EC 2.7.11.1) -
CK060_MOUSE	UPF0360 protein C11orf60 homolog - Mus musculus (Mouse)
SI1L1_RAT	Signal-induced proliferation-associated 1-like protein 1 (SPA-1-like pro
SI1L1_MOUSE	Signal-induced proliferation-associated 1-like protein 1 - Mus musculus
THIM_MOUSE	3-ketoacyl-CoA thiolase, mitochondrial (EC 2.3.1.16) (Beta-ketothiolase)
ATX2_MOUSE	Ataxin-2 (Spinocerebellar ataxia type 2 protein homolog) - Mus musculus
TGM1_RAT	Protein-glutamine gamma-glutamyltransferase K (EC 2.3.2.13) (Transglutam
TGM1_MOUSE	Protein-glutamine gamma-glutamyltransferase K (EC 2.3.2.13) (Transglutam
WISP1_RAT	WNT1-inducible-signaling pathway protein 1 precursor (WISP-1) (ELM-1) -
MEPD_RAT	Thimet oligopeptidase (EC 3.4.24.15) (Endo-oligopeptidase A) (Endopeptid
LZTS2_MOUSE	Leucine zipper putative tumor suppressor 2 - Mus musculus (Mouse)
SHE_MOUSE	SH2 domain-containing adapter protein E - Mus musculus (Mouse)
3BHS7_MOUSE	3 beta-hydroxysteroid dehydrogenase type 7 (3 beta-hydroxysteroid dehydr
TLN2_MOUSE	Talin-2 - Mus musculus (Mouse)
ACES_MOUSE	Acetylcholinesterase precursor (EC 3.1.1.7) (AChE) - Mus musculus (Mouse
OSBL1_RAT	Oxysterol-binding protein-related protein 1 (OSBP-related protein 1) (OR
OSBL1_MOUSE	Oxysterol-binding protein-related protein 1 (OSBP-related protein 1) (OR
RAB28_MOUSE	Ras-related protein Rab-28 - Mus musculus (Mouse)
PRC1_MOUSE	Protein regulator of cytokinesis 1 - Mus musculus (Mouse)

Select Summary Report

Format As | Select Summary (protein hits) | Help

Significance threshold p< 0.05 | Max. number of hits AUTO

Standard scoring ○ MudPIT scoring ◉ | Ions score or expect cut-off 0 | Show sub-sets 0

Show pop-ups ◉ Suppress pop-ups ○ | Sort unassigned Decreasing Score | Require bold red ☐

1. GDF7_MOUSE **Mass:** 47861 **Score:** 53 **Queries matched:** 73 **emPAI:** 0.16

Growth/differentiation factor 7 precursor (GDF-7) - Mus musculus (Mouse)

Query	Observed	Mr(expt)	Mr(calc)	Delta	Miss	Score	Expect	Rank	Peptide
457	841.5600	840.5527	842.4722	-1.9195	1	16	5.2	10	R.EIRAQAR.A
2744	650.9300	1299.8454	1300.6987	-0.8532	0	15	7.6	1	R.AVTASESSPLALR.R
3358	462.2000	1383.5782	1381.6487	1.9294	0	27	0.38	1	R.AAGAGPAWSPGGGGGGR.T 3356
5812	596.4800	1786.4182	1785.0009	1.4172	1	15	5.6	5	R.APGPSALQAAAVPGPRAVR.R
5832	597.4000	1789.1782	1789.8020	-0.6238	0	22	1	3	R.LGFGWPGGGDGGGTAAEER.A 5831

 Created with novaPDF Printer (www.novaPDF.com). Please register to remove this message.

Table 1. Primers for predictive candidate genes

Name	RefSeq ID	Sense primer (5'-3')	Anti-sense primer (5'-3')	Amplicon (bp)
Foxj2	NM_021899.3	CTCTTGAAGTCCCTGTCGGT	CTGCGCCCTGTCTCTGTATA	106
Klf3	NM_008453.5	CCAAAGGAAGCGCAGGATAC	CAGGTGCATTTGTACGGCTT	123
Mef2a	NM_001033713.3	GGGGTGACTTCCATTCTCCA	CATGTGTCCATCCTCATGCG	94
Nobox	NM_130869.3	GCCAACCTTCCTCTTCCTCT	TATGCTGTCACTGCCAGGAA	84
Pou3f1	NM_011141.2	CAAATTTGGGGTGAGGTGGG	TAGGATGGGGAGGGAGAACA	127
Gapdh	NM_008084	ACCCAGAAGACTGTGGATGG	CACATTGGGGGTAGGAACAC	171
Col10α1	NM_009925.4	GCAGCATTACGACCCAAGATC	TCTGTGAGCTCCATGATTGC	201
Runx2	NM_001145920	ACCCAGCCACCTTTACCTAC	TATGGAGTGCTGCTGGTCTG	150

***Foxj2*:** forkhead box J2; ***Klf3/Tef2*:** Kruppel-like factor 3; ***Mef2a*:** myocyte enhancer factor 2A; ***Nobox/OG2*:** NOBOX oogenesis homeobox; ***Pou3f1/Tst-1* :** POU domain, class 3, transcription factor 1; ***Gapdh*:** glyceraldehydes-3-phosphate dehydrogenase; ***Col10α1*:** type X collagen, alpha 1; ***Runx2* :** runt-related transcription factor 2;

Table 2. Primers for candidate genes identified by Y1H

Name	RefSeq ID	Sense primer (5'-3')	Anti-sense primer (5'-3')	Amplicon (bp)
Anxa2	NM_007585.3	CTTCCGAAAGCTGATGGTCG	CACGTCGGTTCCTTTCCTCTT	132
Fn1	NM_001276408.1	TACCCTTCCACACCCCAATC	TGCCAGGAAGCTGAATACCA	122
Hspa5	NM_001163434.1	CCTTGTGTTTGACCTGGGTG	CCATGACCCGCTGATCAAAG	131
Nedd4	NM_010890	CATGTGTGGTCTGGGAGATG	CCAGAACCAGTGGATGACCT	100
Psmb1	NM_011185.3	AGAATGTAGAGCACGTCCCC	ATACACATCCCTCTCGGCTG	89
Ptgs2	NM_011198.3	TGCAGAATTGAAAGCCCTCT	CCCCAAAGATAGCATCTGGA	95
Rab1	NM_008996.3	GCCCTTGAACTCCTGATCCT	GGGCAATCACATTTCCTGCT	123
Rela	NM_009045.4	CACCGGATTGAAGAGAAGCG	AGTTGAGTTTCGGGTAGGCA	132
Rpl35	NM_025592.3	CCTGCGACCCAAGAAGACTA	TCGTTGTCATCTCAGGCCTT	138
Rps28	NM_016844.2	AAATGTCAAAGGCCCCGTTC	TTCCGTGGGCTAAGTAGTGG	122

Anxa2 **:** annexin A2; ***Fn1*** **:** fibronectin 1; ***Hspa5*** **:** heat shock protein 5; ***Nedd4*** **:** neural precursor cell expressed, developmentally down-regulated 4; ***Psmb1*** **:** proteasome (prosome, macropain) subunit, beta type 1; ***Ptgs2*** **:** prostaglandin-endoperoxide synthase 2; ***Rab1:*** member RAS oncogene family; ***Rela:*** v-rel reticuloendotheliosis viral oncogene homolog A (avian); ***Rpl35*** **:** ribosomal protein L35; ***Rps28*** **:** ribosomal protein S28.

Table 3. Primers for candidate genes identified by MS

Name	RefSeq ID	Sense primer (5'-3')	Anti-sense primer (5'-3')	Amplicon (bp)
Ddef2	NM_001004364.2	ATCAAACAGGCCCAGGATGA	TTCCCGTTCCTCTCTGTTCC	179
Foxk2	NM_001080932.2	TGTGCACTTTCAGGTTTCCG	GTTGATGGTCAGGGGTGAGA	137
Fus	NM_139149.2	GAGCTGGAGACTGGAAGTGT	CCCATATGAGAGCCTCCTGG	130
Gdf7	NM_013527.1	ATCGCGCCATTAGACTACGA	GAATGCTGATGGGACTGAGC	175
Hnf3a	NM_008259.3	CTCTCTACCCACCTACGCTG	ACCTCAGCATGACATGACCA	105
N4bp3	NM_145974.3	TATGAGTTCTCCTGCCCCAC	GTCGTTCCACATACAGACGC	169
Rab28	NM_027295.2	TCACACTTCCTCCTGCTCTG	GTGTGGTTCCAAATCGCACT	93
Scrt2	NM_001160410.1	AGCAATTTTGGAGGGTGCTG	TACTGCTGAAGGTGGGGAAG	142
Sfrp4	NM_016687.3	GGAGTGGTTGCAATGAGGTC	TGGCAGGAGGAATTGGTGAT	109
Tgm1	NM_001161714.1	AACAGAAGCACAGATTGGCG	AACCCGAGAGACACTGACTG	139

Ddef2* :** Asap2, ArfGAP with SH3 domain, ankyrin repeat and PH domain 2; ***Foxk2* :** forkhead box K2; ***Fus* :** fused in sarcoma, RNA-binding protein;Gdf7:*** growth differentiation factor 7; ***Hnf3a (Foxa1)* :** forkhead box A1; ***N4bp3* :** NEDD4 binding protein 3; ***Rab28 :*** member RAS oncogene family; ***Scrt2:*** scratch homolog 2, zinc finger protein (Drosophila); ***Sfrp4* :** secreted frizzled-related protein 4; ***Tgm1*:** transglutaminase 1, K polypeptide;
